# The Effect of Cell Salvage on Bleeding and Transfusion Needs in Cardiac Surgery

**DOI:** 10.1155/2022/3993452

**Published:** 2022-09-01

**Authors:** Frixos Tachias, Evangelia Samara, Anastasios Petrou, Agathi Karakosta, Stavros Siminelakis, Efstratios Apostolakis, Petros Tzimas

**Affiliations:** ^1^Department of Anesthesiology and Postoperative Intensive Care, University Hospital of Ioannina, Ioannina, Greece; ^2^Department of Anesthesiology and Postoperative Intensive Care, Faculty of Medicine University of Ioannina, Ioannina, Greece; ^3^Department of Anesthesiology, CH Sud Seine et Marne, Fontainebleau 77310, France; ^4^Department of Cardiothoracic Surgery, University Hospital of Ioannina, Ioannina, Greece

## Abstract

**Introduction:**

Cell salvaging is well established in the blood management of cardiac patients, but there remain some concerns about its effects on perioperative bleeding and transfusion variables. This randomized controlled study investigated the potential effects of the centrifuged end-product on bleeding, transfusion rates, and other transfusion-related variables in adult cardiac surgery patients submitted to extracorporeal circulation.

**Materials and Methods:**

Patients were randomly chosen to receive (cell-salvage group, 99 patients) or not to receive (control group, 110 patients) the centrifuged product of a cell salvage apparatus. Bleeding and transfusion rates according to the universal definition of perioperative bleeding (UDPB) classification, postoperative hemoglobin, coagulation, and oxygenation indices were recorded and compared between the groups.

**Results:**

Both groups had almost identical bleeding and transfusion rates (median value: 2 units of red blood cells (RBC) and no units of fresh frozen plasma (FFP) and platelets (PLT) for both groups, *p* > 0.05). Patients in the cell-salvage group presented slightly higher hemoglobin concentrations (10.6 ± 1.1 vs. 10.1 ± 1.7 g/dL, *p* < 0.05, respectively) and a tendency towards better oxygenation indices (P_a_O_2_/F_i_O_2_: 241 ± 94 vs. 207 ± 84, *p*=0.013) in the postoperative period albeit with a tendency for prolongation of prothrombin time (INR: 1.31 ± 0.18 vs. 1.26 ± 0.12, *p*=0.008).

**Conclusion:**

Within the study's constraints, the perioperative use of the cell salvage concentrate does not seem to affect bleeding or transfusion variables, although it could probably ameliorate postoperative oxygenation in adult cardiac surgery patients. A tendency to promote coagulation disturbances was detected.

## 1. Introduction

Cell salvaging is well established in cardiac surgery [1]. It is an important tool that permits the collection and reinfusion of the patient's own red cells, salvaged from the operating field. This potentially reduces the risks of blood transfusions while maintaining acceptable hemoglobin (Hb) values throughout the intraoperative and early postoperative periods [2, 3]. Apart from its main role in patient blood management [4], there is evidence use of cell salvage is associated with decreased systemic inflammation and a reduced incidence of postoperative atrial fibrillation, a common arrhythmia after cardiac surgery [5]. However, issues of safety and efficacy, and the practical advantages, if any, of “permissive anemia” are still being debated [6, 7].

This single-center, randomized, controlled study aimed to investigate whether cell salvaging can alter the perioperative bleeding and hematologic profiles of adult cardiac surgery patients and, consequently, their transfusion needs.

## 2. Materials and Methods

We prospectively investigated more than 200 adult patients submitted to cardiac surgery at the University Hospital of Ioannina. Recruitment took place with approval from the hospital's ethics committee (decision No: 14/7-7-2015 – *θ*.10, Ethics committee, Scientific Council, University Hospital of Ioannina, Ioannina, Greece).

### 2.1. Inclusion and Exclusion Criteria

The inclusion criteria consisted of the patient's written informed consent to join the study, age > 18 years, and cardiac surgery (coronary bypass surgery, valve surgery, aortic replacement surgery, or mixed surgery) with extracorporeal circulation (ECC) lasting > 90 minutes. Exclusion criteria comprised failure of in-time anticoagulant medication discontinuation, emergency cases, and surgery without at least 90 minutes of ECC minutes (on- or off-pump surgery, pericardial effusion drainage, pacemaker manipulations). Institutional guidelines for preoperative antithrombotic management consist of a 5-day discontinuation of clopidogrel, continued aspirin intake up to the eve of surgery, and the last intake of Low Molecular Weight Heparin (LMWH) 8–12 hours preoperatively. Only those patients with unstable angina who, under maximal anti-ischemic treatment, met the anticoagulant's discontinuation criteria were included in the study.

### 2.2. Group Size Calculation

A priori power analysis indicated that the study-group population should exceed 98 recruits per group to achieve a power of 80% and a statistically significant level of 5% of an estimated decrease in anticipated transfusion rate of 13% between groups with a given transfusion incidence at 45 ± 15% for the control group [8].

### 2.3. Randomization of Recruits

All patients were randomly assigned to either intraoperative cell salvaging (study group, group CS) or allogeneic red cell transfusions according to the center's transfusion policy, analyzed below (control group, group C). At admission to the operating theater, the nurse anesthetist blindly drew a closed, randomizing envelope. To allow for dropout cases, the cards included a 15% surplus.

In the preoperative visit, after obtaining the patient's informed consent to join the study, one investigator collected basic demographic and somatometric information, retrieved the necessary EuroScore II data, and verified compliance with inclusion and exclusion criteria.

### 2.4. Anesthesia Management

In the operating theater, the monitoring application, intravenous arterial access, pulmonary artery catheterization, basic tympanic temperature, and induction technique followed institutional standards. Following the insertion of an arterial catheter, the baseline blood samples for blood gas analysis and for hematological and biochemical profiling were obtained. Before beginning the operation, a bolus dose of tranexamic acid (5 mg/kg) was administered, followed by a continuous infusion (5.5 mg/kg/h) until the end of the operation. Intraoperative anticoagulation was affected by an initial heparin dose of 400 iu/kg i. v. and additional doses as needed to achieve and maintain the Activated Clotting Time (ACT) target value of 480 sec. [9].

### 2.5. Cell Salvage Management

The Haemonetics Cell Saver® 5 device was used in this study, equipped with a 2 liter reception reservoir, a cylindrical filter of 150 *μ*m, and a centrifugation bowl of 225 ml. For all patients in Group CS, the following automatic mode settings were applied: 5650 rpm centrifugation speed, 200–500 ml/min washing rate with normal saline, and a washing volume of 1000 ml per bowl.

The cell salvage reservoir collected lost blood from the moment of pericardiotomy to the ECC, and after ECC weaning to the end of the surgery. During ECC, the operating field suction (“pump sucker”) returned all blood aspirates into the ECC reservoir. In CS patients, the remaining pump volume limited to 1000 ml was directed to the cell salvage reservoir at the end of ECC. As soon as it was available, the cell salvage concentrate was transfused to the CS-group patients either in the operating room or in the postoperative intensive care unit (ICU).

### 2.6. Transfusion Protocol

The center's transfusion policy consisted of the following:

(i) RBC (1 unit) when Hb < 8.5 g/dL together with SvO_2_ < 60% in patients with CO > 2.5 L/min/m^2^ and bedside recheck
(ii) In the presence of active bleeding after heparin reversal:
  1. Fibrinogen concentrate (2–4 gr) if blood fibrinogen < 200 mg/dL.
  2. FFP (2–4 units) or PCC (up to 2500 iu) if INR > 1.5 (bedside check).
  3. PLT (5 units) if PLT < 100 × 10^9^/*μ*L.

### 2.7. Statistical Analysis

Distribution normality was evaluated using the Kolmogorov–Smirnov test. According to the normality result, student's *t*-test or its nonparametric equivalent, the Mann–Whitney *U*-test, was used to compare continuous variables between the two groups studied. Fisher's exact test or the *χ*^2^ test was employed to compare categorical variables, as appropriate.

Comparisons of cell salvage specimens before (A) and after centrifugation (B) were conducted using single-factor repeated-measures ANOVA. The Bonferroni correction was applied for comparisons of patients' perioperative hematologic parameters between the groups.

Data values are presented either as mean ± standard deviation for continuous normally distributed variables, as medians for nonnormally distributed data, or as frequencies for categorical data. All tests were two-tailed and subject to a significance threshold of 5% (*p* < 0.05). Data were analyzed using ΙΒΜ® SPSS® 32.

## 3. Results

### 3.1. Recruitment

**Scheme 1:**
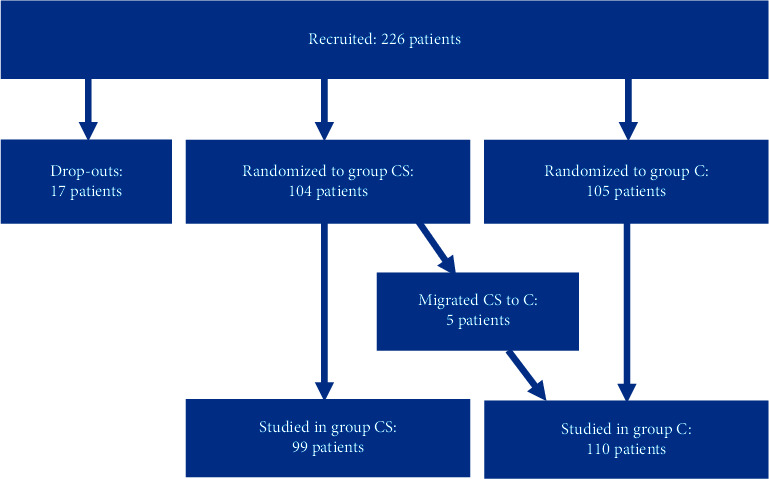
Recruitment and randomization of patients in cell salvage (CS) and control (C) groups.

By the end of the study, 226 patients had been recruited (Scheme 1). The dropout rate was 7.5% (17 patients). The following were the main reasons for leaving the study: actual duration of ECC was less than 90 minutes, 10 patients (5 in group CS, 5 in group C); last-minute decision was taken to perform off-pump bypass surgery instead of on-pump, 5 patients (3 in group CS, 2 in group C); and failure of sufficient postoperative data collection, 2 patients (1 for group CS, 1 for group C). Five patients of the cell-salvage group did not receive the collected blood as their hemodynamics and the oxygen delivery-consumption equilibrium were adequately assured by intraoperative RBC transfusions due to delays in preparing the centrifuged product. Consequently, these 5 patients were transferred to group C.

In the end, we studied 209 patients; 99 who received intraoperative salvaged blood and 110 who received homologous blood transfusion without intraoperative salvaging.

### 3.2. Demographic and Intraoperative Data

The study population was homogenous regarding demographic characteristics and comorbidities (Table 1). Group CS exhibited a statistically significant prolongation in the duration of ECC (10 minutes) and of the corresponding aortic cross clamping (CCX) time (7 minutes) compared with group C (Table 2). No differences were detected in fluid administration (volume and type of solutions), nor in urine output (2).

### 3.3. Quantitative and Qualitative Characteristics

The characteristics of the salvaged blood before (A) and after centrifugation (B) are presented in Table 3.

The concentration effect achieved is expressed in the statistically significant increase of the Hb concentration and the corresponding hematocrit values. Specifically, our apparatus and settings produced a concentration effect for hemoglobin of 150%.

### 3.4. Bleeding, Transfusions, and Coagulation Disorders

**Figure 1:**
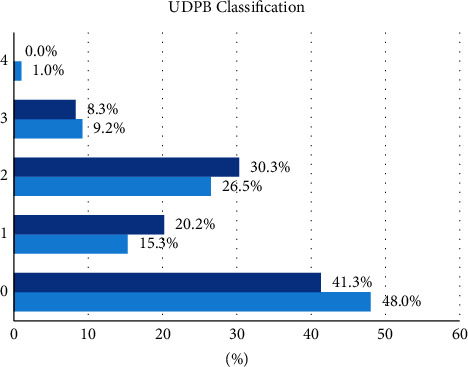
Perioperative universal definition of perioperative bleeding (UDPB) classification (^(*f*)^: fisher's exact test, ^(*x*)^: *χ*^2^ test).

The total volume of thoracic drains at 24 h and at removal (Table 4), as well as the classification of perioperative bleeding according to UDPB [10], was similar between the groups (Figure 1).

**Figure 2:**
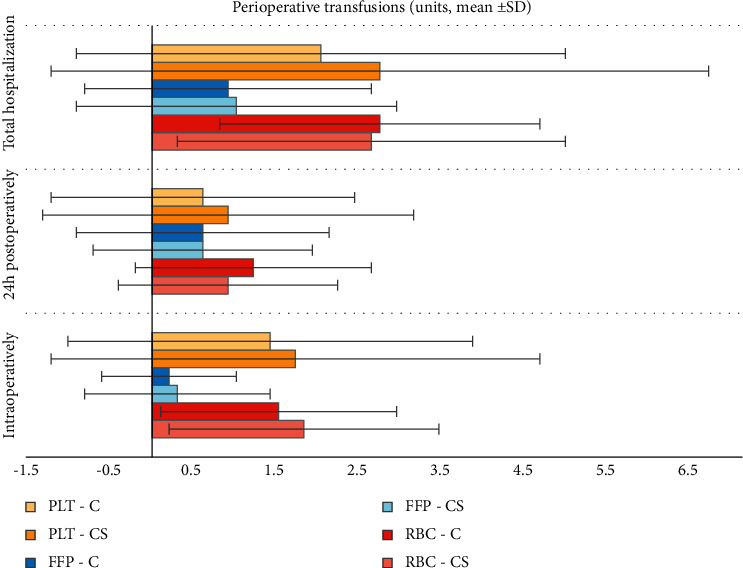
Perioperative transfusions (FFP: fresh frozen plasma, PLT: platelets, RBC: red blood cells, ^(*f*)^: fisher's exact test).

The overall perioperative (including intraoperatively till 24 h postoperatively) mean transfusion volume received by both groups did not differ significantly (2.7 ± 1.9 vs. 2.6 ± 2.3 RBC units, *p* > 0.05, 0.9 ± 1.7 vs. 1.0 ± 1.9 FFP units, *p* > 0.05 and 2.0 ± 2.9 vs. 2.7 ± 3.9 PLT units, *p* > 0.05 for groups C and CS, respectively), while the median values were 2 units of RBC and no units for FFP and PLT for both groups (Figure 2). Consequently, there were no significant differences in the UDPB classification between the groups.

The mean volume of cell salvage concentrate transfused in group CS patients was 715 ± 278 ml (median volume: 724 ml, range: 238–1574 ml). The net result of the cell salvage concentrate transfusion was that at 24 h, the Hb concentration in group C was significantly lower than that in group CS (10.1 ± 1.7 vs. 10.6 ± 1.1 g/dL, *p* < 0.05, respectively). Approximately the same difference was also detected in the minimum Hb value, from the time of admission to the postoperative unit and 24 hours postoperatively (9.1 ± 1.3 vs. 9.5 ± 0.9 gr/dL, *p* < 0.05, median values: 9.2 vs. 9.4 g/dL for the C and CS groups, respectively, 4).

In each group, 3 patients received prothrombin complex concentrates (PCC, 1000–2000 i.u.) intraoperatively according to our coagulation management protocol, while 8 patients in group C and 6 in group CS received 5–10 mg of vitamin K after weaning from ECC. The fibrinogen concentrate and PCC, as well as the tranexamic acid, DDAVP, and calcium chloride administrations, were comparable between the groups (Table 2).

In terms of postoperative coagulation disorders, we did not detect significant differences between the groups, apart from a statistically higher INR ratio at the time of admission in the postoperative care unit and at 24 h postoperatively in the cell salvage group (Table 4).

## 4. Discussion

The study shows that administering the concentrate from the cell salvage device, operated with the settings used, did not significantly reduce the amount of allogenic transfusion in our patients.

### 4.1. Bleeding Associated with Cell Salvaging

The perioperative bleeding data exhibited no differences between groups concerning the patient's UDPB bleeding classification, the amounts of blood products transfused perioperatively, and the percentages of reoperation (“take back”) or patients transfused intraoperatively. The difference in mean postoperative hemoglobin concentrations at 24 h and in its lowest postoperative values of only 0.5 g/dL in favor of the cell-salvage group patients could be attributed to the relatively low concentration effect, with an Hct of approximately 42% vs. Hct > 50% that is reported in the manufacturers' datasheet [11]. This, together with a shortened lifespan of salvaged RBCs [12, 13], might have mutually contributed to comparable transfusions of RBCs between the groups.

Certainly, this is not a new observation. A number of studies in the past decade [14–16], and the most recently published meta-analysis, showed that cell salvaging did not affect the volume of RBCs transfused and also implied a tendency (though not statistically significant) for increased use of FFPs and PLTs in the cell-salvage group [8, 16]. On the contrary, another meta-analysis of 2018 revealed that cell salvaging decreases the percentage of patients transfused perioperatively as well as the volume of allogenic blood products transfused [3].

### 4.2. Coagulation Factors and Clinical Indices

Our infused concentrate had low platelet and fibrinogen concentrations and an undetectable INR (INR > 10) and APTT (>180 sec). A lowering of platelet count was revealed, at the time of ICU admission, in both groups, in contrast to other techniques (HemoSep© ultra-filtration) [17] that discovered an augmenting PLT content and preserved the thromboelastography-evaluated platelets' functionality [18]. The Cardiotomy Trial revealed prolongation of INR and thrombin time and reductions of fibrinogen levels for at least 12 h postoperatively [19], while other authors described a consumption coagulopathy in the cell salvaging group [14] and a significant decrease of various coagulation factors (I, II, VII, XI, XIII) in the cell salvage concentrate [20]. Due to activated coagulation, combined with accelerated fibrinolysis and postoperative bleeding in their cell-salvage group, some investigators called for careful use of cell-salvage techniques in patients at high risk for perioperative bleeding [14], while others revealed higher costs [21] and recommended avoiding retransfusion volumes above 1 liter [22], despite the recommendation for routine use by various scientific societies [22–26]. All these effects may certainly explain our results concerning the prolonged INR ratios of our patients at 24 h after admission, albeit without increased thoracic drain output.

### 4.3. Oxygenation Indices

The study detected a significantly better mean PaO_2_/FiO_2_ ratio in the patients that received the cell-salvage concentrate. The development of postoperative hypoxia in cardiac surgery patients is multifactorial, but the receipt of RBC or FFP perioperatively is frequently reported as an important triggering factor irrespective of their potential to produce circulation overload and transfusion-related acute lung injury (TRALI) [27]. Notably, in our study, at 16–24 h and 24–32 h postoperatively, significantly fewer patients in the cell-salvage group had their lowest PaO_2_/FiO_2_ between 100 and 200 compared with those in the control group (49.4 vs. 63.8% and 35.3 vs. 51.8%, respectively). These ratios, among many other causative factors, might well imply a TRALI, but, immediately after cardiac surgery, this diagnosis remains challenging and uncertain.

### 4.4. Limitations

There are some limitations to consider. The durations of ECC and aortic cross clamp were longer for the cell salvage group compared with the control group, but the difference of 10 minutes between groups is probably not clinically significant enough to seriously challenge the randomization benefits of this prospective study. The dose of tranexamic acid incorporated into our protocol is considered a “medium dose scheme” [28, 29], which did not lead to increased bleeding that could obscure a potential positive effect of cell salvaging in bleeding and transfusion patterns [30]. Due to laboratory issues, we could not measure the heparin content, the coagulation factors, or perform thromboelastic and platelet function tests in our samples in order to elucidate their pro- or anticoagulant profiles.

## 5. Conclusions

Using the Haemonetics Cell Saver® 5 device, in the auto mode setting used, did not demonstrably affect the transfusion patterns of our patients. Its concentrate is poor in platelets, fibrinogen and probably all the other coagulation proteins. Its transfusion slightly raised the Hb concentration but did not affect the percentage of patients transfused with allogenic blood products, nor the volume of units transfused. To the contrary, it might have positively affected patients' oxygenation. We think that better-performing modes or devices that assure a higher Hb concentration in the end product could demonstrate more favorable transfusion patterns.

## Figures and Tables

**Table 1:** Demographics and comorbidities (^(*x*)^: *χ*^2^ test, ^(*u*)^: Mann–Whitney *U*-test, ^(*f*)^: fisher's exact test).

Parameter	Group CS mean ± SD, *n*, or (%)	Group C mean ± SD, *n*, or (%)	*p*
Number of patients	99	110	
EuroSCORE II	2.45 ± 2.3	2.12 ± 1.6	0.24^(*x*)^
Age	66.28 ± 10	67.06 ± 10	0.58^(*u*)^
Gender “male”	74 (76%)	87 (79%)	0.62^(*x*)^
Gender “female”	25 (24%)	23 (21%)	0.62^(*x*)^
BSA, (m^2^)	1.9 ± 0.6	1.9 ± 0.3	0.92^(*u*)^
Creatinine clearance (ml/min)	73.4 ± 25.6	74.9 ± 26.2	0.77^(*u*)^
Peripheral vascular disease	(19.19%)	(25.45%)	0.41^(*x*)^
Decreased mobility	(6.06%)	(2.73%)	0.31^(*f*)^
Re-operation	(0.0%)	(1.82%)	0.50^(*f*)^
C.O.P.D.	(12.12%)	(20.0%)	0.14^(*x*)^
Active endocarditis	(1.01%)	(0.0%)	0.47^(*f*)^
Critical state	(1.01%)	(0.0%)	0.47^(*f*)^
I.D.D.M.	(10.0%)	(6.36%)	0.32^(*f*)^

NYHA classification			
NYHA I	(11.11%)	(6.36%)	0.23^(*f*)^
NYHA II	(47.47%)	(57.27%)	0.15^(*x*)^
NYHA III	(39.39%)	(35.45%)	0.55^(*x*)^
NYHA IV	(2.02%)	(0.91%)	0.56^(*f*)^
Unstable angina	(13.13%)	(10.91%)	0.67^(*x*)^

Left ventricular contractility			
Good contractility, EF > 50%	(61.62%)	(61.82%)	1.00^(*x*)^
Moderate contractility, EF: 30–50%	(35.35%)	(33.73%)	0.88^(*x*)^
Poor contractility, EF < 30%	(3.03%)	(4.45%)	0.70^(*f*)^
Recent M.I.	(29.29%)	(14.55%)	0.88^(*x*)^

Pulmonary hypertension			
Moderate (SPAP 35–59 mmHg)	(25.25%)	(14.55%)	0.07^(*x*)^
Severe (SPAP > 60 mmHg)	(2.02%)	(0.0%)	0.49^(*f*)^
Emergency operation	(0.0%)	(0.0%)	1.00^(*f*)^

Surgery			
Only CABG	(61.62%)	(63.63%)	0.78^(*x*)^
One non-CABG operation	(25.26%)	(29.09%)	0.64^(*x*)^
Two operations	(10.00%)	(7.27%)	0.35^(*f*)^
Three operations	(1.01%)	(0.0%)	0.47^(*f*)^
Thoracic aorta operation	(6.06%)	(2.73%)	0.31^(*f*)^

**Table 2:** Perioperative fluid balance, extracorporeal circulation characteristics, and administration of coagulation products (^(*t*)^: student's *t*-test, ^(*u*)^: Mann-Whitney *U*-test, ^(*x*)^: *x*^2^ test).

Intraoperative data	Group CS mean ± SD, (%)	Group C mean ± SD, (%)	*p*
Total fluids intraoperatively (ml)	3362.6 ± 1390.4	3107.2 ± 1077.5	0.149^(*t*)^
Urine before ECC (ml)	128.7 ± 127.8	123.6 ± 130.5	0.778^(*u*)^
Urine during ECC (ml)	925.2 ± 834.7	743.7 ± 419.7	0.053^(*u*)^
Urine after ECC (ml)	518.2 ± 313.6	540.9 ± 304.4	0.606^(*u*)^
Duration of ECC (min)	135.5 ± 35.1	125.8 ± 29.3	**0.033** ^(*u*)^
Duration of aortic cross clamp (min)	88.7 ± 29.1	81.4 ± 21	**0.042** ^(*u*)^
Lower temperature during ECC (°C)	31.3 ± 1.4	31 ± 1.4	0.144^(*u*)^
Colloids intraoperatively (ml)	505.1 ± 50.3	509.2 ± 67.4	0.615^(*t*)^
Fibrinogen intraoperatively (g)	0.7 ± 1.0	0.7 ± 0.9	0.843^(*u*)^
PCC intraoperatively (IU/kg)	30.3 ± 223.8	46.7 ± 287.6	0.646^(*u*)^
Tranexamic acid (mg)	3398 ± 1186.2	3036.7 ± 1499.2	0.056^(*u*)^
DDAVP (IU)	0.56 ± 4.0	0.93 ± 5.6	0.586^(*u*)^
CaCl_2_ (mg)	878.8 ± 539.7	749.5 ± 531.4	0.085^(*u*)^
“Take back”	(4.1%)	(3.7%)	1.000^(*x*)^

The bold values indicate significantly different at *p* < 0.05.

**Table 3:** Cell salvage specimens' analysis (A: before centrifugation, B: after centrifugation, ^(*a*)^: ANOVA repetitive measures test).

Parameter	Sample A mean ± SD	Sample B mean ± SD	*p*
Volume (ml)	2119 ± 768	715 ± 278	**0.001** ^(*a*)^
Hb (g/dL)	5.9 ± 1.5	14.8 ± 2.3	**0.001** ^(*a*)^
Hct (%)	17.1 ± 4.43	42.4 ± 6	**0.001** ^(*a*)^
WBC (*n*^×9^/L)	5.66 ± 3.21	13.06 ± 6.16	**0.001** ^(*a*)^
Neutrophils (%)	71.5 ± 10.2	81 ± 8	**0.016** ^(*a*)^
RBC (*n*^×12^/L)	2.04 ± 0.56	5.08 ± 0.81	**0.001** ^(*a*)^
PLT (*n*^×9^/L)	83 ± 39.8	16.43 ± 10.01	**0.001** ^(*a*)^
Fibrinogen (mg/dL)	126 ± 54	42 ± 13	**0.001** ^(*a*)^

The bold values indicate significantly different at *p* < 0.05.

**Table 4:** Perioperative hematologic parameters and total thoracic drains (^(*t*,*b*)^: student's *t*-test with Bonferroni correction).

Parameter	Hb (g/dL)		Platelets (*n*^×9^/L)		INR ICU		aPTT (sec)		Fibrinogen (mg/dL)		PaO_2_/FiO_2_		Thoracic drains 24 h (ml)		Thoracic drains total (ml)		Minimum Hb (g/dL)	
Group	CS	C	CS	C	CS	C	CS	C	CS	C	CS	C	CS	C	CS	C	CS	C
Preoperatively mean ± SD	12.9 ± 1.7	13.4 ± 1.4	250 ± 87	229 ± 69	1.13 ± 0.3	1.07 ± 0.1	31.8 ± 3.9	32.4 ± 4.9	409 ± 108	399 ± 98								
*p* between groups	0.069^(*t*,*b*)^		0.055^(*t*,*b*)^		0.805^(*t*,*b*)^		0.202^(*t*,*b*)^		0.548^(*t*,*b*)^									

At ICU admission mean ± SD	10.3 ± 1.2	10.1 ± 1.2	141 ± 50	147 ± 50	1.36 ± 0.17	1.32 ± 0.14	38.3 ± 6.5	37.4 ± 8.1	259 ± 58	265 ± 67	307 ± 104	310 ± 112						
*p* between groups	0.102^(*t*,*b*)^		0.498^(*t*,*b*)^		0.049^(*t*,*b*)^		0.341^(*t*,*b*)^		0.669^(*t*,*b*)^		0.692^(*t*,*b*)^							

24 h postoperatively mean ± SD	10.6 ± 1.1	10.1 ± 1.7	130 ± 51	136 ± 49	1.31 ± 0.18	1.26 ± 0.12	42.6 ± 8.5	40.6 ± 9.5	462 ± 81	454 ± 98	241 ± 94	207 ± 84	667 + 388	660 + 445				
*p* between groups	**0.013 ** ^(*t*,*b*)^		0.363^(*t*,*b*)^		**0.013** ^(*t*,*b*)^		0.065^(*t*,*b*)^		0.491^(*t*,*b*)^		**0.008** ^(*t*,*b*)^		0.683^(*t*,*b*)^					

Hospitalisation mean ± SD															981 ± 571	924 ± 929	**9.5** ± **0.9**	9.1 ± 1.3
*p* between groups															0.607^(*t*,*b*)^		**0.013 ** ^(*t*,*b*)^	

The bold values indicate significantly different at *p* < 0.05.

## Data Availability

All data are available upon reasonable request from the corresponding author.
